# Editorial: Robots that Learn and Reason: Towards Learning Logic Rules from Noisy Data

**DOI:** 10.3389/frobt.2021.755933

**Published:** 2021-09-16

**Authors:** Plinio Moreno, Alexandre Bernardino, José Santos-Victor, Rodrigo Ventura, Kristian Kersting

**Affiliations:** ^1^ Institute for Systems and Robotics, Instituto Superior Técnico, Universidade de Lisboa, Lisbon, Portugal; ^2^ Centre for Cognitive Science, Computer Science Department, TU Darmstadt, Darmstadt, Germany

**Keywords:** learning logic rules, robotics, predicate grounding, two-level planning, reinforcement learning

From the early developments of AI applied to robotics by Hart et al. (1968), Duda and Hart (1972) and Lozano-Pérez and Wesley (1979), higher level commands were grounded to real world sensing by carefully design algorithms, which provide a link between the abstract predicates and the sensors and actuators. In order to have fully autonomous robots that learn by exploration and by imitation, the grounding algorithms between the higher-level predicates and the lower-level sensors and actuators should be discovered by the robot. Previous and recent efforts on robotics aim to discover and/or learn these intermediate layer commands, which must cope with discrete and continuous data. The main objective of this Research Topic is to advance on learning logic rules from noisy data. We have four articles that address: Logic rules that cope with states that are not directly observable by the sensing modalities; learning rules that represent object properties and their functionalities, which are grounded to the particular robot experience; learning low-level robot control actions that fulfill a set of abstract predicates in a two-level planning approach; learning to develop skills in a robotic playing scenario by composing a set of behaviors. In the following, we introduce the four articles and their contributions to rule learning in presence of noisy data.

The article by Zuidberg Dos Martires et al. introduces a general-purpose semantic object tracker, which anchors sensory input to object representations in a probabilistic manner. The semantic tracker is able to estimate the object position in the presence of total occlusions of the object by using a multi-modal probability distribution, which is modeled as a distributional logic tree. In addition, the tree structure of the multi-modal distribution is estimated from observations, which provides a set of data-driven clauses that can be augmented with recursive ones. This methodology allows to model objects that can be occluded by objects that are themselves occluded.

The article by Thosar et al. defines measurable object properties and their functionalities, focusing on the robot-centered estimation of the object properties for multiple categories. Regarding the object properties, the robot executes actions that interact with the objects, measuring the effects on the objects through proprioceptive changes in the robot and external sensors. The robot-centric conceptual knowledge is obtained from unsupervised clustering of the numerical values, where each cluster represents a qualitative property (i.e., concept). Regarding the object functionalities, these are selected according to a set of predefined tasks, where a quantitative value of the functionality is obtained from vision-based sensors. The object properties and their functionalities are designed for selecting a tool substitute, which is addressed as a classification problem. The learning algorithm selects tool substitutes very similar to human experts, showing the validity of the approach. This methodology allows to select tool substitutes in a robot-centric manner, based on unsupervised discovery of qualitative object properties.

The article by Eppe et al. proposes a methodology that integrates an abstract (high-level) planner using Planning Domain Definition Language (PDDL), and an actor-critic Deep Neural Network (low-level) using Hindsight Experience Replay (HER). On one hand, model-free Reinforcement Learning (RL) with Universal Value Function Approximators (UVFAs) and HER provide the tools to build a model that grounds a discrete symbolic action space in continuous action trajectories. The hybrid nature of the approach allows to cope with complex casual dependencies, such as stacking three blocks in experimental conditions with realistic noise. On the other hand, although the predicates that correspond to the discrete symbolic space are handcrafted properly for the scenarios, the low-level subgoal *g*_*sub*_ works across predicates. This design allows to have only one RL model that executes actions for all the created predicates. This hybrid two-level planner works better than the more general Hierarchical Reinforcement Learning (HRL) for the ant navigation scenario, but the application to other domains requires always the handcrafted predicate design.

The article by Hangl et al. proposes a methodology that improves previously developed work on skill learning, which relies on composition of a fixed set of simple behaviors. The main contribution of this article is to discover the sequence (i.e., composition) of simple behaviors that leads to the goal, which in previous work was set by an expert. The behaviors are learnt in a bottom-up hierarchical fashion by running the sensing → preparation → basic behavior execution → reward loops, using the Projective Simulation (PS) framework that includes: 1) actions for sensing, 2) perceptual states and 3) preparatory behaviors. The basic building block of PS is a clip, which creates connections between the perceptual states and the behaviors. On top of the clips, a skill is defined as a behavior or a composition of behaviors that lead to successful execution. To acquire new skills, a random walk through the PS framework is performed by hopping from clip to clip until a behavior is reached. The robot learns by adapting the probabilities of a clip given the previously executed clip, according to the received reward. Once a behavior is reached with a high accuracy, a new behavior is explored as continuation of the previous one. Complex manipulation behaviors are discovered by the model, such as book grasping and tower disassembling. However, the behavior compositions are limited to perceptual states of the same sensing behavior.

This research topic contributes to the very challenging problem of understanding the world from perceptual input while acquiring predicates that describe the task-environment relations. The selected articles provide insights on rule learning for: 1) Non-observable states during visual tracking, 2) representation of objects and their properties in a robot-embodied way, 3) developing skills in a robotic playing scenario, and 4) low-level robot control actions that fulfill predicates in a two-level planning approach.
